# Regulation of IGF -1 signaling by microRNAs

**DOI:** 10.3389/fgene.2014.00472

**Published:** 2015-01-13

**Authors:** Hwa Jin Jung, Yousin Suh

**Affiliations:** ^1^Department of Genetics, Albert Einstein College of MedicineNew York, NY, USA; ^2^Department of Medicine, Albert Einstein College of MedicineNew York, NY, USA; ^3^Institute for Aging Research, Diabetes Research and Training Center, Albert Einstein College of MedicineNew York, NY, USA

**Keywords:** IGF-1 signaling, microRNAs, aging, aging-related disease, therapeutic agents

## Abstract

The insulin-like growth factor 1 (IGF-1) signaling pathway regulates critical biological processes including development, homeostasis, and aging. Dysregulation of this pathway has been implicated in a myriad of diseases such as cancers, neurodegenerative diseases, and metabolic disorders, making the IGF-1 signaling pathway a prime target to develop therapeutic and intervention strategies. Recently, small non-coding RNA molecules in ∼22 nucleotide length, microRNAs (miRNAs), have emerged as a new regulator of biological processes in virtually all organ systems and increasing studies are linking altered miRNA function to disease mechanisms. A miRNA binds to 3’UTRs of multiple target genes and coordinately downregulates their expression, thereby exerting a profound influence on gene regulatory networks. Here we review the components of the IGF-1 signaling pathway that are known targets of miRNA regulation, and highlight recent studies that suggest therapeutic potential of these miRNAs against various diseases.

## INTRODUCTION

**FIGURE 1:**
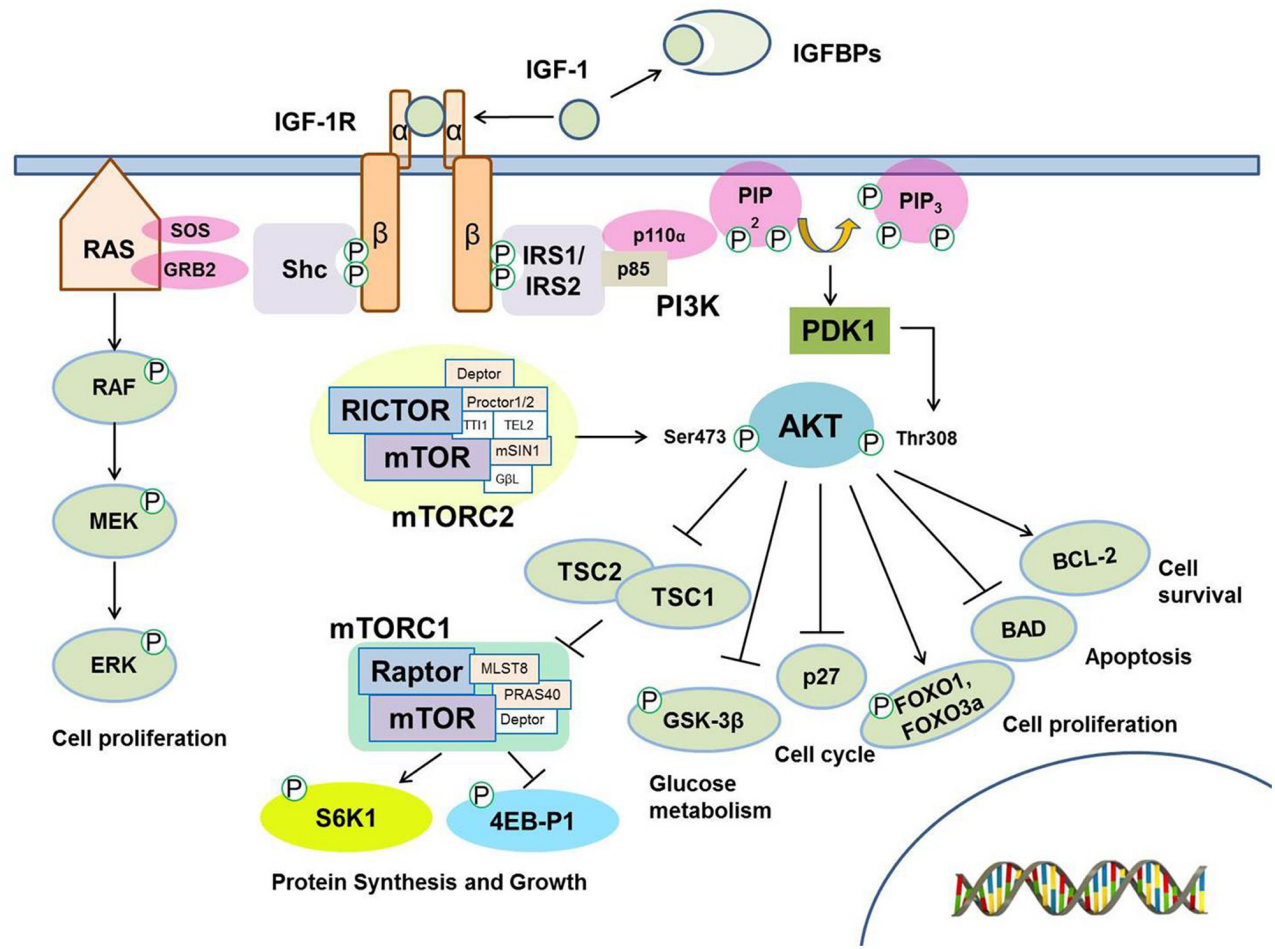
**The insulin growth factor 1 (IGF-1) signaling pathway.** IGF binding proteins (IGFBPs) modulate IGF -1 bioavailability. IGF -1 functions as a ligand to interact with IGF -1 receptor (IGF -1R) in the cellular membrane, which leads to autophosphorylation and recruitment of the adaptor proteins IRS-1, IRS-2, and Shc. The interaction of IRS-1 and IRS-2 with IGF -1R induces the activation of the class I phosphatidyl inositol 3’ kinase (PI3K). PI3K converts PIP2 to the lipid second messenger PIP3. AKT family of kinases is activated by PDK1 and by mTOR-containing complex mTORC2 resulting in the phosphorylation at Threonine 308 (Thr308) and Serine 473 (Ser473), respectively. Activated AKT then regulates downstream signaling molecules including Tuberous sclerosis protein 1/2 (TSC1/2) which inhibit mTORC1 complex and regulate S6K1/2 and 4EB-P1 phosphorylation, FOXO transcription factors, GSK-3β, p27, BAD, and BCL-2. These downstream molecules are involved in several cellular processes including protein synthesis, glucose metabolism and cell survival. In parallel, Shc activation induces the activation of the RAS/MAP kinase pathway, which results in increased cell proliferation.

The insulin-like growth factor 1 (IGF-1) signaling pathway is a highly conserved regulatory module that coordinates growth, development, and metabolism. It regulates multiple cellular processes including proliferation, differentiation, energy metabolism, glucose homeostasis (Chen et al., 2010; Smith-Vikos and Slack, 2012). The IGF-1 signaling pathway is comprised of ligands, IGF binding proteins (IGFBPs) that modulate ligand availability, transmembrane receptors, and downstream signaling and effector molecules (**Figure 1**; Zha and Lackner, 2010). IGF-1 binds to insulin-like growth factor 1 receptor (IGF-1R), a heterotetrameric transmembrane receptor tyrosine kinase (RTK) comprised of two alpha and two beta subunits. Binding of the ligand to IGF-1R leads to receptor activation through autophosphorylation of IGF-1R. Activation of IGF-1R results in the recruitment and phosphorylation of multiple adaptor proteins including insulin receptor substrates (IRSs) and Shc, leading to activation of two pro-survival signaling pathways. Phosphorylation of IRS-1 or IRS-2 activates phosphoinositol 3-kinase (PI3K)-PDK1-AKT signaling pathway, while phosphorylation of Shc leads to activation of RAS, RAF and extracellular signal-regulated kinase (ERK)/mitogen-activated protein kinase (MAPK) signaling pathway. Activation of AKT through phosphorylation at Threonine 308 by PDK1 or at Serine 473 by mTORC2 promotes cell survival by multiple mechanisms, including inhibition of apoptosis and induction of pro-survival gene expression. AKT signaling also influences glucose metabolism by regulating GSK-3β activity and protein synthesis and cell growth by regulating the activity of the mTORC1 complex (Efeyan and Sabatini, 2010). Phosphorylation of GSK3β by AKT blocks its activity, leading to the dephosphorylation and activation of the eukaryotic translation initiation factor 2B (eIF2B) involved in protein synthesis and cell survival (Welsh et al., 1998). AKT phosphorylation inhibits Tuberous sclerosis protein 2 (TSC2), a GTPase activating protein (GAP), leading to activation of mTOR1 complex followed by activation of S6K. This results in release of 4EB-P1 from eIF4E which can then direct ribosomes to the cap structure of mRNAs to promote cap-dependent translation, one of major functions of the IGF-1R axis (Tognon and Sorensen, 2012). The other parallel pathway, the RAS-RAF-MAPK, stimulates cell proliferation (Pollak, 2008).

Dysregulation of the IGF-1 signaling pathway has been implicated in a variety of aging-related diseases including muscle disease, cardiovascular diseases (CVDs), neurodegenerative diseases, metabolic diseases, and cancer (Andreassen et al., 2009; Zha and Lackner, 2010; Zemva and Schubert, 2011; O’Neill et al., 2012; Piccirillo et al., 2014). In addition, it is one of the major conserved pathways of aging that regulate lifespan in model organisms across a great evolutionary distance from *Caenorhabditis elegans* (Kimura et al., 1997), *Drosophila* (Tatar et al., 2001), and to mice. Reduced signaling of the IGF1 pathway has been implicated in human longevity (Tazearslan et al., 2011; Milman et al., 2014) as in model organisms. Therefore, there has been a great deal of attention toward development of therapeutic intervention targeting the IGF-1 signaling pathway (Yuen and Macaulay, 2008). Clinical trials are now ongoing with directed targeting of IGF signaling against advanced solid tumors such as non-small cell lung cancer (NSCLC; Karp et al., 2009; Ramalingam et al., 2011).

**FIGURE 2:**
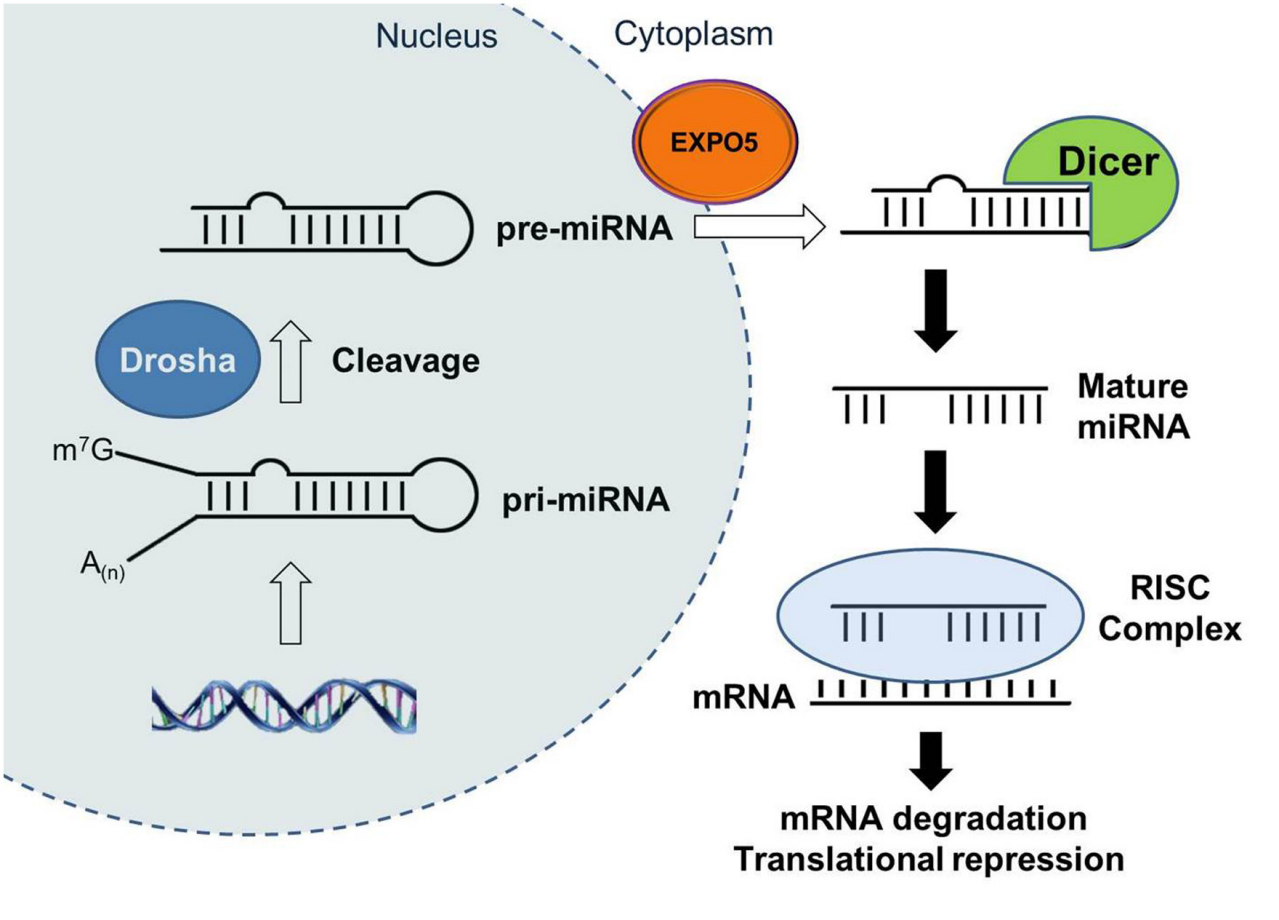
**The biogenesis of microRNAs.** MicroRNAs (miRNAs) are initially transcribed by polymerase II (Pol II) as primary-miRNA (pri-miRNA) transcripts which are processed by Drosha to generate pre-miRNAs. Pre-miRNAs are exported from nucleus to cytoplasm by exportin 5 (EXPO5). The Dicer complex is recruited to pre-miRNAs to remove the stem loop from pre-miRNAs, and then mature miRNAs which is one strand of the miRNA duplex are incorporated into RNA-induced silencing complex (RISC). Within the RISC, miRNAs bind to complementary sequences of target mRNAs to repress their translation or induce their degradation.

Recently, microRNAs (miRNAs) have emerged as a new regulator of critical biological processes and shown to be involved in disease mechanisms, including cancer, cardiovascular, and neurodegenerative disease (Saugstad, 2010; Small and Olson, 2011). miRNAs are small non-coding RNAs in ∼18–25 nucleotides (nt) length that regulate gene expression at the post-transcriptional level. They are initially transcribed as primary-miRNA (pri-miRNA) with a stem–loop structure and following cleavage by the RNase III enzyme Drosha become precursor miRNA (pre-miRNA; **Figure 2**). The pre-miRNAs are then exported from nucleus into the cytoplasm by exportin 5 (EXPO5). In cytoplasm, the RNase III enzyme Dicer cleaves the pre-miRNA to generate intermediate double strand RNA duplex (Jung and Suh, 2012, 2014; Smith-Vikos and Slack, 2012). The ∼22-nt long mature miRNA strand obtained from the intermediate duplex is then loaded into the Argonaute-containing RNA-induced silencing complex (RISC) and target mRNA molecules by miRNA:mRNA sequence complementarity and negatively affects gene expression either through mRNA cleavage and degradation via the perfect base-pairing with target mRNA or translation repression via imperfect complementation with 3′UTR of target mRNA (Llave et al., 2002; Smith-Vikos and Slack, 2012). A miRNA can target multiple mRNAs and one mRNA can be controlled by multiple miRNAs (Hobert, 2008). Predicted to target up to 1/3rd of the human genome (Lewis et al., 2005), miRNAs profoundly impact gene regulatory networks and influence the important physiological and pathological processes. In addition, in contrast to other cellular mediators, miRNAs can be easily manipulated and therapies based on antimiRs or miRNA mimics are now being developed to repress pathological miRNAs (Elmen et al., 2008; Lanford et al., 2010; Small and Olson, 2011) or to overexpress protective miRNAs (Small and Olson, 2011), respectively. Despite the pervasive role of miRNAs in essential biological processes and as promising therapeutic targets, there is only a few dedicated reviews on their role in regulation of the IGF-1 signaling pathway (Lee and Gorospe, 2010). Here we provide a comprehensive review focused on the components of the IGF-1 signaling regulated by miRNAs (**Table 1**) and discuss their potential role as therapeutic agents.

**Table 1:** Insulin-like growth factor-1 signaling pathway-targeting miRNAs.

IGF-1 signaling pathway	Targeting microRNA	Disease	Model	Species	Reference
IGF-1	Let-7f	Rett syndrome	*Mecp2* KO mice	Mouse	Mellios et al. (2014)
	miR-1	Cardiac hypertrophy	Mouse neonatal cardiomyocytes	Mouse	Elia et al. (2009)
	miR-98	Alzheimer’s disease	APP/PS1 mice and N2a cell line	Mouse	Hu et al. (2013)
	miR-486	Lung cancer	Non-small cell lung cancer tissues	Human	Peng et al. (2013)
IGF1R	Let-7	Diabetes	Transgenic mice	Mouse	Zhu et al. (2011)
	miR-7	Tongue squamous cell carcinoma	Tongue squamous cell carcinoma cell lines	Human	Jiang et al. (2010)
	miR-16	Osteosarcoma	Osteosarcoma tissues and cell lines	Human	Chen et al. (2013)
	miR-99a	Psoriasis	Psoriasis skin tissues and keratinocytes	Human	Lerman et al. (2011)
		Oral squamous cell carcinoma	Oral squamous cell carcinoma tissues and cell lines	Human	Yen et al. (2014)
	miR-122	Breast cancer	MCF7 cells	Human	Wang et al. (2012)
	miR-145	Hepatocellular carcinoma	Tumorous liver tissues	Human	Law et al. (2012)
		Bladder cancer	T24, 5637, and TCHu169 cells	Human	Zhu et al. (2014)
	miR-148a	Breast cancer	Breast cancer cell lines	Human	Xu et al. (2013)
	miR-152	Breast cancer	Breast cancer cell lines	Human	Xu et al. (2013)
	miR-182	Muscle aging	Skeletal muscle tissues and myocytes	Human	Olivieri et al. (2014)
	miR-195	Lung cancer	Non-small cell lung cancer tissues and A549, H157, H1975, and Calu-3 cells	Human	Wang et al. (2014b)
	miR-223	Epithelial carcinoma	HeLa cells	Human	Jia et al. (2011)
		Muscle aging	Skeletal muscle tissues, myocytes, and MCF7 cells	Human	Olivieri et al. (2014)
	miR-378	Cardiac remodeling	Heart tissues and cardiomyocytes	Mouse	Knezevic et al. (2012)
	miR-383	Glioma	Glioma cancer tissues and cell lines	Human	He et al. (2013)
	miR-470	Ames dwarf and GHRKO mice	Brain tissues and WI-38 cells	Mouse	Liang et al. (2011)
	miR-486	Lung cancer	Non-small cell lung cancer tissues and H460, H1299, and A549 cells	Human	Peng et al. (2013)
	miR-497	Cervical cancer	Cervical cancer tissues and cell lines	Human	Luo et al. (2013)
		Colorectal cancer	HCT116 cells	Human	Guo et al. (2013)
	miR-515-5p	Breast cancer	Ashkenazi Jewish DNA and breast cancer cell lines	Human	Gilam et al. (2013)
	miR-669b	Ames dwarf and GHRKO mice	Brain tissues and WI-38 cells	Mouse	Liang et al. (2011)
	miR-675	Development	Embryonic tissues and cell lines	Mouse	Keniry et al. (2012)
	miR-681	Ames dwarf and GHRKO mice	Brain tissues and WI-38 cells	Mouse	Liang et al. (2011)
IRS-1	miR-145	Colon cancer	Colorectal cancer cell lines	Human	Shi et al. (2007)
		Hepatocellular carcinoma	Tumorous liver tissues	Human	Law et al. (2012)
		Bladder cancer	T24, 5637, TCHu169 cells	Human	Zhu et al. (2014)
	miR-148a	Breast cancer	Breast cancer cell lines	Human	Xu et al. (2013)
	miR-152	Breast cancer	Breast cancer cell lines	Human	Xu et al. (2013)
IRS-2	Let-7	Diabetes	Transgenic mice	Mouse	Zhu et al. (2011)
	miR-145	Hepatocellular carcinoma	Tumorous liver tissues	Human	Law et al. (2012)
					*(Continued)*
PIK3R1 (p85a)	miR-486	Lung cancer	Non-small cell lung cancer	Human	Peng et al. (2013)
PIK3R3	miR-193a-5p	Lung cancer	Non-small cell lung cancer tissues and cell lines	Human	Yu et al. (2014)
AKT	miR-100	Dermal wound healing	Mouse dermal wound healing model	Mouse	Jin et al. (2013)
RICTOR	miR-34a	Glioma	Glioma stem cell lines	Human	Rathod et al. (2014)
	miR-218	Oral cancer	Oral squamous cell carcinoma cell lines	Human	Uesugi et al. (2011)
mTOR	miR-99a	Cervical cancer	Cervical cancer tissues and HeLa cell line	Human	Wang et al. (2014a)
	miR-99b	Cervical cancer	Cervical cancer tissues and HeLa cell line	Human	Wang et al. (2014a)
	miR-193a-5p	Lung cancer	Non-small cell lung cancer tissues and cell lines	Human	Yu et al. (2014)
S6K2	miR-193a-3p	Lung cancer	Non-small cell lung cancer tissues and cell lines	Human	Yu et al. (2014)
FOXO1	miR-370	Prostate cancer	Prostate cancer cell lines	Human	Wu et al. (2012)
FOXO3a	miR-96	Breast cancer	Breast cancer tissues and cell lines	Human	Lin et al. (2010)
	miR-223	Muscle aging	Skeletal muscle tissues, myocytes, and MCF7 cells	Human	Olivieri et al. (2014)

*APP, amyloid precursor protein; PS1, presenilin 1; GHRKO mice, mice with targeted deletion of the growth hormone receptor.*

## THE KEY COMPONENTS OF THE IGF-1 SIGNALING PATHWAY TARGETED BY microRNA REGULATION

### INSULIN-LIKE GROWTH FACTOR 1

Insulin-like growth factor 1 is a classical circulating hormone that is expressed in many tissues, especially in liver, indicating that apart from the endocrine action, its autocrine and paracrine function is a significant component of IGF-1 action. Unlike other growth factors, IGF-1 acts as both a mitogen and a differentiation factor involved in the mitogenic and myogenic processes during muscle development, regeneration, or hypertrophy. Cardiac hypertrophy has been inversely correlated with the expression of miR-1 (Care et al., 2007; Sayed et al., 2007) and miR-1 depleted mice show cardiac defects, including misregulation of cardiac morphogenesis, electric conduction, and cell proliferation (Yang et al., 2007; Zhao et al., 2007). In investigating the role of miR-1 in cardiac and skeletal muscle, it was shown that miR-1 expression level was decreased in cardiac hypertrophy mouse, while IGF-1 protein level was significantly increased, as compared to control (Care et al., 2007; Sayed et al., 2007; Elia et al., 2009; Lee and Gorospe, 2010). It was shown that miR-1 bound to IGF-1 3′UTR directly and controlled its expression level. Consistently, when miR-1 was overexpressed in skeletal muscle cells, IGF-1 signaling was markedly reduced as measured by significant reduction in phosphorylation of both FOXO3a and AKT.

Insulin-like growth factor 1 has an important role in brain function such as neuroendocrine secretion and cognitive function (O’Neill et al., 2012). Particularly, IGF-1 accelerates Aβ clearance from the brain. Dysregulation of IGF-1-mediated signaling has been associated with Alzheimer’s disease (AD; Hong and Lee, 1997; Vargas et al., 2011). miR-98 has shown to increase in AD mouse model (Wang et al., 2009) and miR-98 was found to target IGF-1 3′UTR directly leading to the decreased IGF-1 expression at both mRNA and protein levels (Hu et al., 2013). In addition, the level of Aβ42 was increased by miR-98 overexpression in the cell lysates of AD cell culture model (N2a/APP) while IGF-1 supplementation rescued the Aβ42 accumulation, suggesting that miR-98 is involved in AD pathogenesis by negatively regulating IGF-1.

Insulin-like growth factor 1 has been demonstrated to play an important role in Rett syndrome, a severe childhood onset neurodevelopmental disorder caused by mutations in methyl-CpG-binding protein 2 (*MECP2*). Administration of IGF-1 to *Mecp2* KO mice rescued disease-related symptoms (Castro et al., 2014). Clinical trials of recombinant human IGF-1 for Rett syndrome have reported to be safe (Pini et al., 2012). A recent study implicates a role of miRNA in Rett syndrome through regulation of IGF-1 (Mellios et al., 2014). In this study, treatment of β2-adrenergic receptor agonist clenbuterol improved the behavior of *Mecp2* KO mice in terms of survival, respiratory deficiency and motor coordination. It was found that clenbuterol treatment decreased let-7f expression level, which was robustly increased in the cerebellum of *Mecp2* KO mice. let-7f was shown to bind directly to 3′UTR of IGF-1 and dysregulated IGF-1 mRNA level, which was rescued by clenbuterol treatment. These data suggest that *Mecp2*-mediated let-7f upregulation leads to IGF-1 depletion in Rett syndrome, implicating let-7f as a potential therapeutic target for Rett syndrome.

Insulin-like growth factor 1 dysregulation has been reported in many cancers including NSCLC (Scagliotti and Novello, 2012). Peng et al. (2013) has investigated the role of miRNAs in lung cancer focused on the IGF-1 signaling pathway as a therapeutic target. They performed a high throughput miRNA array analysis using a cohort of stage 1 adenocarcinomas and found that miR-486 was the most downregulated miRNA in lung tumors compared to adjacent uninvolved lung tissues. miR-486 was shown to bind directly to IGF-1 3′UTR decreasing IGF-1 mRNA and protein levels in NSCLC cell lines (Peng et al., 2013). Consistently, miR-486 overexpression in different lung cancer cell lines reduced cell growth and migration. Furthermore, nude mice injected with NSCLC cells overexpressing miR-486 showed no detectable tumors and decreased IGF-1 level, while control mice formed xenograft tumors. This study demonstrates that miR-486 functions as tumor suppressor by targeting IGF-1 in NSCLC.

### INSULIN-LIKE GROWTH FACTOR 1 RECEPTOR

Since, the initial discovery that mutations in daf-2, an IGF-1R homolog, extend lifespan in *C. elegans*, there is overwhelming evidence that dampening of IGF-1 signaling increases longevity in model organisms from worms to mice (Kenyon, 2010). Recently, miRNAs have been shown to regulate life span of *C. elegans* by targeting, in part, the components of the IGF-1 signaling pathway including daf-2 (Smith-Vikos and Slack, 2012). To investigate potential role of miRNAs in longevity, Liang et al. (2011) performed high throughput profiling of miRNAs in brain tissues of the two long-lived mutant mice, Ames dwarf and GHRKO mice. They found three miRNAs, miR-470, -669b, and -681, were upregulated in these mice and these three miRNAs directly bound to IGF-1R 3′UTR, downregulated IGF-1R at both mRNA and protein levels, and led to reduced phosphorylation of both AKT and FOXO3a. These results suggest that miRNAs may contribute to longevity in mice through downregulation of IGF-1R as in *C. elegans*.

Insulin-like growth factor 1 pathway is an important pathway in muscle mass regulation by activating protein synthesis and inhibiting protein degradation (Velloso, 2008). Recently, Olivieri et al. (2014) has investigated the effect of miRNAs on the IGF-1 signaling pathway in skeletal muscle under estrogen hormone treatment. The post-menopausal women using hormone replacement therapy (HRT) have been reported to have ∼5% greater muscle strength than those not using HRT (Greising et al., 2009). miRNA profiles of *vastus lateralis* muscle samples of nine healthy monozygotic female twin pairs (54–62-years old) discordant for HRT showed that miR-182 and miR-223 were significantly downregulated in HRT treated groups as compared to non-treated groups (Olivieri et al., 2014). In MCF7 cell culture model, estrogen treatment also reduced the expression levels of miR-182 and miR-223 and increased IGF-1 signaling. Among the predicted targets of miR-182 and miR-223, IGF-1R and FOXO3a were validated as their direct targets suggesting that HRT-mediated dysregulation of miR-182 and miR-223 induces the activation of IGF-1 signaling pathway by regulating IGF-1 signaling genes including IGF-1R and FOXO3a.

Insulin-like growth factor 1 receptor level has been known to decrease in postnatal cardiac remodeling (Cheng et al., 1995). Knezevic et al. (2012) has investigated the role of miRNAs as a mechanism of decreased IGF-1R level during the postnatal period. They tested the expression level of 23 randomly selected miRNAs in the mouse neonatal heart (7 days after birth) and fetal heart at 16 days gestation. miR-378 was significantly increased with more than 10-fold in neonatal heart as compared to fetal heart and was a highly abundant miRNA in the heart. In this study, they found that miR-378 bound to IGF-1R 3′UTR directly and miR-378 overexpression enhanced apoptosis of cardiomyocytes with decreased IGF-1 signaling activity suggesting that miR-378 has a role in postnatal cardiac remodeling and cardiomyocyte survival against stressors by negatively regulating IGF-1R.

Keniry et al. (2012) has investigated the role of *H19* large intergenic non-coding RNA (lincRNA) which is known to be most highly abundant and conserved transcripts in mammalian cells. In this study, miR-675 embedded in *H19* was expressed in the placenta but suppressed in the embryonic tissues. Based on the target prediction analysis, they found that miR-657 indeed bound to IGF-1R 3′UTR and miR-675 overexpression reduced the cell proliferation in embryonic and extra-embryonic cell lines, suggesting that miR-675 is involved in the *H19*-mediated developmental control by targeting IGF-1R.

Psoriasis has been known to be a common chronic inflammatory skin disorder (Galadari et al., 2005). Lerman et al. (2011) has investigated the mechanism of psoriasis focusing on miRNAs since the involvement of miRNAs in skin development has been reported in mouse model (Yi et al., 2006). In this study, miR-99a expression level was decreased in lesion skin and uninvolved skin as compared to normal skin (Lerman et al., 2011). Since, IGF-1R has been known to be involved in the pathogenesis of psoriasis and upregulated in psoriasis, they studied the relationship between miR-99a and IGF-1R. miR-99a bound to 3′UTR of IGF-1R directly and negatively regulated IGF-1R mRNA and protein expression levels. Interestingly, miR-99a-overexpressing human keratinocyte cells showed slower proliferation as compared to the control cells suggesting that miR-99a may function in the development of psoriasis by targeting IGF-1R.

The let-7 miRNA family members have been demonstrated to function as tumor suppressors by targeting the oncogenes and cell cycle regulators (Kumar et al., 2008). Zhu et al. (2011) investigated the roles of let-7 miRNA in mammalian glucose metabolism. They used inducible let-7 transgenic mice and measured the metabolic changes such as glucose tolerance and signaling activity and found that inducible let-7 transgenic mice showed decreased glucose tolerance. To investigate the mechanism of let-7 effect on glucose metabolism, they overexpressed let-7 in C2C12 myoblasts and measured the PI3K-mTOR signaling activities. In addition, they found that let-7 bound directly 3′UTR of IGF-1R as well as IRS-2 and insulin receptor (INSR) suggesting that let-7 may function as a regulator of glucose metabolism by targeting multiple components of growth/metabolic signaling pathway genes.

Insulin growth factor 1 receptor-mediated pro-oncogenic signaling has been demonstrated in multiple cancers and molecular mechanisms leading to dysregulation of IGF-1R has been an intense focus of cancer research. IGF-1R protein is overexpressed in more than 40% of breast cancer (BC; Tamimi et al., 2011). Xu et al. (2013) has investigated whether miR-148a and miR-152 are involved in the regulation of IGF-1R-mediated signaling activity in BC. In this study, they found that both miR-148a and miR-152 are downregulated in BC cell lines due to DNA hypermethylation as compared to normal breast cells. Both miR-148a and miR-152 were shown to directly bind to 3′UTR of IGF-1R and inhibit IGF-1R-mediated PI3K/AKT activation. Cell proliferation was suppressed in BC cell lines stably overexpressing miR-148a or miR-152, while BC cells lacking IGF-1R 3′UTR binding sites of miR-148a and miR-152 restored cell proliferation. These results suggest that miR-148a and miR-152 affect cell growth via IGF-1R regulation. Recently, Wang et al. (2012) has reported that miR-122 involves in BC. miR-122 has been well-known to play a role in liver physiology and to be suppressed in primary hepatocellular carcinoma (HCC) suggesting that miR-122 has a tumor suppressor role (Bai et al., 2009). The authors have shown that the expression of miR-122 in human BC cells and specimens is reduced, while their IGF-1R expression levels were increased (Wang et al., 2012). From the luciferase binding assay, they found that miR-122 directly bound to IGF-1R and regulated IGF-1R-mediated signaling pathway. Furthermore, the effect of miR-122 on inhibition of cell proliferation could be reversed by IGF-1R overexpression demonstrating that miR-122 has a role as a tumor suppressor in BC through the IGF-1R inhibition. miR-515-5p has been also shown as a regulator of IGF-1R in BC (Gilam et al., 2013). Interestingly, this study showed single nucleotide polymorphisms (SNPs) within miRNA binding sites of IGF-1R affected BC risk in *BRCA1* mutation carriers. They performed genotyping of the A/G SNP (rs28674628) in the 3′UTR of the *IGF1R* gene for 115 Ashkenazi Jewish BC patients, all carriers of the 185delAG *BRCA1* mutation and found that all *BRCA1* carriers harboring the G allele of the rs28674628 SNP were diagnosed with BC by age 45 years, whereas almost 50% of the wild type (A) allele homozygotes were BC free at that age. The IGF-1R targeting of miR-515-5p was disrupted by A to G nucleotides substitution in the miR-515-5p binding sites of IGF-1R 3′UTR, suggesting that the IGF-1R level in BC can be regulated by miR-515-5p binding on the polymorphic site in IGF-1R 3′UTR.

Following up on the study showing that that miR-7 is decreased in advanced tongue squamous cell carcinoma (TSCC; Liu et al., 2009a,b), Jiang et al. (2010) has investigated IGF-1R as a potential target of miR-7 in TSCC. In this study, miR-7 was shown to directly bind to IGF-1R and reduced its protein as well as mRNA levels in TSCC cell lines. In addition, they demonstrated that miR-7-mediated downregulation of IGF-1R attenuated the IGF-1-induced activation of AKT and led to reduced cell proliferation and cell cycle arrest, and to an increase in apoptosis rate. This study suggests that miR-7 regulates TSCC cell growth, at least in part, by targeting IGF-1R. miR-7 has been known to function as tumor suppressor in several human cancers and target a number of proto-oncogenes, including IRS1, IRS2 and epidermal growth factor receptor (EGFR; Kefas et al., 2008). In oral squamous cell carcinoma (OSCC), IGF-1R has been known to be overexpressed in OSCC tissues and cell lines (Brady et al., 2007) and one of predictors of clinical outcome in patients with OSCC (Lara et al., 2011; Yen et al., 2014). Recently, Yen et al. (2014) has shown that IGF-1R is one of direct targets of miR-99a in OSCC, which has been reported in several studies to be downregulated in clinical samples of OSCC in different stages (Chen et al., 2012; Yan et al., 2012). miR-99a expressing OEC-M1 cells showed decreased migration and invasion activities and these effects were rescued by ectopic expression of IGF-1R indicating that miR-99a has a role as a tumor suppressor in OSCC via IGF-1R regulation.

miR-145 has been reported to target IGF-1R in several cancer studies (Law et al., 2012; Zhu et al., 2014). miR-145 level was downregulated, while IGF-1R level was upregulated, in human urinary bladder transitional cell carcinoma cell lines (T24, 5637) compared to normal bladder epithelial cells (TCHu169; Zhu et al., 2014). They found that overexpression of miR-145 led to inhibition of IGF-1-induced cell proliferation and miR-145 was shown to directly bind to 3′UTR of IGF-1R and IRS-1 in the bladder cancer cells. These data suggest that miR-145 plays a role as a tumor suppressor by targeting IGF-1R and IRS-1 in bladder cancer. miR-145 was also shown to modulate IGF-1 signaling pathway by directly targeting IGF-1R in HCC (Law et al., 2012). In this study, the expression level of miR-145 was found to be downregulated in a cohort of 80 HCC cases as compared to the adjacent non-malignant liver tissues. Consistently, miR-145 overexpression in HKCI-C2 cells induced G2-M arrest and apoptosis suggesting that miR-145 has the repressive effect of hepatic malignant growth through negative regulation of IGF-1R. miR-223 has been known to be repressed in HCC cells as compared with normal liver tissue (Su et al., 2009), but its role other cancer remained unknown (Laios et al., 2008). Jia et al. (2011) has made miR-223 overexpression model in ovarian cancer cell line and found that miR-223 remarkably suppressed the proliferation, growth rate and colony formation. miR-223 injected nude mice also showed the inhibition of tumor formation. In this study, miR-223 was shown to directly bind to IGF-1R and repress IGF-1 signaling cascade including AKT/mTOR/p70S6K suggesting that miR-223 functions as tumor suppressor through the suppression of IGF-1R-mediated cell growth signaling.

Amplified IGF-1/IGF-1R signaling has been associated with increased relative risk for development of colorectal cancer (CRC) as well as CRC metastasis, and resistance to chemotherapeutic drugs (Weber et al., 2002; Sekharam et al., 2003). Guo et al. (2013) has investigated the role of miR-497 in aberrant expression of IGF-1R in CRC. miR-497 expression was significantly decreased in both CRC tissues CRC cell lines as compared to normal mucosa from miRNA array analysis. miR-497 was found to target IGF-1R 3′UTR and downregulate PI3K/AKT signaling activities. In addition, miR-497 overexpression in CRC cells led to inhibition of cell proliferation and invasive behavior thereby increasing the sensitivity of CRC cells to apoptosis induced by anti-cancer drugs such as CDDP and 5-FU. Interestingly, in this study, downregulation of miR-497 was associated with the reduction of copy number in a specific fragment of chromosome shown in ∼71% of colon cancers suggesting that miR-497 may have a role as tumor suppressor in CRC. Same with human cervical cancer, miR-497 targeted IGF-1R directly resulting in the decrease of IGF-1R mRNA and protein levels and suppressed migration and invasiveness of cervical cancer suggesting that miR-497 functions as tumor suppressor in human cervical cancer by post-transcriptionally targeting IGF-1R (Luo et al., 2013).

Recently, Wang et al. (2014b) has reported that miR-195 expression was downregulated in NSCLC clinical tissue and cell lines and inhibited NSCLC cell proliferation. They found that miR-195 could bind to IGF-1R directly and has a tumor suppressive effect, which was attenuated by IGF-1R expression. In addition, miR-486 was shown to have a potent tumor suppressor role in NSCLC through direct targeting of IGF-1R as well as IGF-1 (Peng et al., 2013).

He et al. (2013) has investigated tumor suppressive miRNAs in glioma. They analyzed the published microarray-based, high throughput microRNA expression dataset (GSE25631) to find dysregulated miRNAs. Among them, miR-383 was significantly downregulated in human glioma tissues as compared to normal brain tissue. miR-383 overexpression induced the inhibition of glioma cell invasion and miR-383 inhibition promoted glioma cell invasion. They found that miR-383 targeted IGF-1R 3′UTR and negatively regulated the expression of IGF-1R which resulted in decreased IGF-1R and AKT signaling activity. Cell invasion promoted by miR-383 inhibition was rescued by IGF-1R inhibition suggesting that miR-383 functions as tumor suppressor by targeting IGF-1R directly and regulating IGF-1R-mediated cell invasion in glioma.

Chen et al. (2013) has investigated the role of miR-16 in osteosarcoma (OS). miR-16 expression level was known to be downregulated in OS tissues compared to healthy bone tissue by miRNA microarray analysis (Jones et al., 2012). Chen et al. (2013) has shown that miR-16 overexpression in OS cells inhibited OS cell proliferation while anti-miR-16 transfected OS cells promoted cell growth. They found that miR-16 targeted IGF-1R 3′UTR directly suggesting that miR-16 functions as tumor suppressor in OS by targeting IGF-1R and regulating cell proliferation.

### INSULIN RECEPTOR SUBSTRATE

Insulin receptor substrate-1 and -2 are two of the major substrates of IGF-1R. IRS-1 and -2 have important roles in cell growth and cell proliferation (Baserga, 2000). Dysregulation of IRS-1 and -2 has been found in many types of cancer (Cantarini et al., 2006; DeAngelis et al., 2006). miR-145 has been shown to regulate IRS-1 in colon cancer (Shi et al., 2007) and IRS-1/2 in HCC (Law et al., 2012). Shi et al. (2007) has investigated the role of miR-145 in colon cancer cell lines including HCT116 and DLD1. miR-145 overexpression induced downregulated IRS-1 protein level but not in the cells without IRS-1 3′UTR. In this study, miR-145 overexpression also inhibited cell growth in HCT116 cell lines suggesting that miR-145 functioned as a tumor suppressor by negatively regulating IRS-1 in colon cancer cells. In HCC, miR-145 directly targeted IRS-1 and IRS-2 by binding 3′UTR of them. Law et al. (2012) has shown that miR-145 overexpression repressed the expression level of IRS-1 and IRS-2 and decreased β-catenin activity thereby resulting in decreased cell growth.

Xu et al. (2013) has shown that miR-148a and miR-152 target IRS-1 as well as IGF-1R in BC. They investigated if miR-148a and miR-152 inhibited PI3K/AKT pathway via targeting IRS-1 using IRS-1 loss- and gain-of-function experiments in BC cells. They found that the knockdown of endogenous IRS-1 significantly decreased PI3K/AKT signaling and this effect was similarly shown in BC cells with miR-148a or miR-152 overexpression leading to the inhibition of cell proliferation. Consistently, deletion of miR-148a or miR-152 binding regions in 3′UTR of IRS-1 did not exert the effect of miR-148a or miR-152 overexpression on inhibition of cell proliferation, indicating that these miRNAs affect cancer cell proliferation via IRS-1 suppression.

Let-7 was also shown to target IRS-2 by direct binding to its 3′UTR as well as those of IGF-1R and INSR (Zhu et al., 2011). As previously described under the subsection of IGF-1R, the authors investigated the let-7 function in glucose metabolism using inducible let-7 transgenic mice model and found that let-7 overexpression induced glucose intolerance by targeting multiple components of the insulin-PI3K-mTOR signaling pathway including IRS-2, IGF-1R, and INSR.

### PHOSPHOINOSITIDE-3-KINASE, REGULATORY SUBUNIT (PIK3R)

Recent study has been demonstrated that miR-193a-5p targeted PIK3R3 by binding its 3′UTR directly in NSCLC (Yu et al., 2014). Yu et al. (2014) investigated the role of miRNAs in NSCLC metastasis and generated miRNA profiles of SPC-A-1sci (high metastatic) and SPC-A-1 (weakly metastatic) cells. Among differentially expressed miRNAs, miR-193a-5p was significantly downregulated in human NSCLC as compared to non-cancerous lung tissue. They also tested its effect on cell proliferation and epithelial–mesenchymal transition (EMT). SPC-A-1sci cells stably expressing miR-193a-5p showed suppressed rates of migration with inhibited cell proliferation, while SPC-A-1sci expressing miR-193a-5p inhibitors enhanced migration and proliferation, as compared to control cells. The same effects were observed in terms of EMT demonstrating that miR-193a-5p inhibited the EMT of NSCLC cells. They tested if miR-193a-5p effect was induced by targeting PIK3R3 using PIK3R3 siRNA. When they treated si-PIK3R3 in NSCLC cells, the enhanced migration and invasion by miR-193a-5p inhibitors were attenuated suggesting that PIK3R3 is a direct and functional target of miR-193a-5p in NSCLC. Targeting PIK3R3 by miR-193a-5p suppressed phosphorylation of AKT (Ser473). Another miRNA shown to target PIK3R1 in NSCLC is miR-486 (Peng et al., 2013). miR-486 overexpression resulted in decreased phosphorylation of PIK3CA, AKT (Ser473), and FOXO3a and induced cell cycle arrest and reduced migration in NSCLC.

### AKT

Jin et al. (2013) has investigated the role of miRNAs in wound healing. They performed microRNA expression profiling analysis on skin samples of unwounded mice, and skin biopsy samples harvested at 1 and 5 days post-wounding. They found that miR-99 family including miR-99a/b and miR-100 was significantly downregulated on day 1 and returned to basal level on day 5. Overexpression of miR-100 suppressed the cell proliferation and cell migration and reduced IGF-1-induced signaling activity by dephosphorylation of p70S6K and 4EB-P1. miR-100 bound directly to AKT1 leading to reduced AKT1 mRNA and protein levels. These data suggest that miR-99 family, especially miR-100 regulates the wound healing process by targeting AKT1 and AKT/mTOR signaling pathway which is a major factors in cell migration and proliferation that contribute to the replenishment of tissues after injury.

### RICTOR

RICTOR is a component of the mammalian target of rapamycin complex 2 (mTORC2). This complex directly controls the phosphorylation of AKT at Ser 473 and promotes cell growth (Hresko and Mueckler, 2005). Uesugi et al. (2011) has shown that miR-218 targets RICTOR by binding its 3′UTR and functions as a tumor suppressor in OSCC. They identified tumor suppressor-miRNAs silenced by DNA hypermethylation in OSCC and their targets using function-based screening with a cell proliferation assay. Among them, miR-218 was downregulated in OSCC cell lines and primary tumor samples, which correlated with hypermethylation in miR-218 promoter region. Either miR-218 to overexpression or knock-down of RICTOR by specific siRNA in OSCC cells led to reduced phosphorylation of AKT suggesting that miR-218 act through the TOR-AKT signaling pathway by targeting RICTOR in OSCC.

Recently Rathod et al. (2014) has reported miR-34a as a RICTOR targeting miRNA in glioma stem cells. miR-34a expression level was downregulated in glioma tissue samples compared to normal tissue samples and in HNGC-2 glioma cells compared to non-tumorigenic neural stem cell-line HNGC-1. When miR-34a was overexpressed in HNGC-2 cells, cell proliferation and cell cycle progression were decreased, while caspase-dependent apoptosis was increased, as compared to control cells. In addition, they injected empty vector (EV)-expressing cells or miR-34a-overexpressing cells to NOD/SCID mice and monitored tumor growth for 45 days. They found that miR-34a-overexpressing mice showed significantly reduced tumors as compared to EV-injected mice suggesting that miR-34a functioned as a tumor suppressor *in vivo*. They tested if miR-34a overexpression affects signaling activity and found that miR-34a overexpression in glioma stem cells profoundly decreased levels of p-AKT (Ser473) and increased GSK-3β levels. Taken together, this data suggest that miR-34a targets RICTOR and thereby regulates AKT/mTOR pathway which causes pronounced effects on glioma malignancy.

### mTOR

mTOR is a serine/threonine protein kinase that regulates growth, proliferation, and survival and a component of both mTORC1 and mTROC2. mTOR 3′UTR was shown to be targeted by miR-99a/b in cancer cell lines. miR-99a/b has been associated with tumor pathogenesis and development of several types of human cancers such as renal cell carcinoma (Cui et al., 2012), HCC (Li et al., 2011), and NSCLC (Kang et al., 2012). Wang et al. (2014a) has investigated miR-99a/b function in human cervical cancer. They measured miR-99a/b expression level in primary and metastatic patients with cervical cancer and found that both of miRNAs are downregulated in primary lesions with and without lymphatic metastasis suggesting that miR-99a/b acts in metastasis of cervical cancer. In this study, miR-99a/b overexpression inhibited cell proliferation and invasion of cervical cancer cells whereas miR-99a/b knockdown by antisense oligonucleotides (ASOs)-based inhibition showed attenuated effect. From the miRNA target prediction, they selected mTOR as a candidate target and found that mTOR 3′UTR bound directly to miR-99a/b using luciferase binding assay. mTOR expression level was decreased by miR-99a/b expression in cervical cancer cell line such as HeLa cells. When they treated HeLa cells with rapamycin to inhibit mTOR activity, they found that the cell proliferation and invasion were significantly inhibited. However, this effect of rapamycin was reversed when they transfected ASO-miR-99a/b suggesting that miR-99a/b functions as tumor suppressor to inhibit the proliferation and invasion of cervical cancer through the targeting of mTOR pathway. Another miRNA shown to target mTOR is miR-193a-5p involved in regulation of migration and invasion *in vitro* and metastasis *in vivo* in NSCLC (Yu et al., 2014).

### S6K

The ribosomal protein S6K (S6 kinase) is a major effector of the mTORC1 and controls fundamental cellular processes including translation, protein and lipid synthesis, cell growth, and cell metabolism (Yu et al., 2014). Yu et al. (2014) has showed that S6K2 were upregulated in NSCLC and S6K2 protein level was associated with lymph node metastasis. In this study, miR-193a-3p was shown to target S6K2 directly, leading to reduced cell proliferation and invasion in NSCLC cells. Conversely, miR-193a-3p inhibition promoted tumorigenic effect, which could be diminished by S6K2 knock-down, suggesting that miR-193a-3p has a role in tumor suppression in NSCLC through the targeting of S6K2.

### FOXO

The FOXO subfamily represents evolutionarily conserved transcription factors that play a critical role in a various biological processes including apoptosis, cell cycle and, DNA repair (Greer and Brunet, 2005). FOXO is often dysregulated in cancer. FOXO3a has been reported to be negatively regulated by miR-96 in human BC (Lin et al., 2010). In the study, miR-96 was found to be overexpressed in BC cell lines and BC tissues (Lin et al., 2010). When co-overexpressed with FOXO3a with 3′UTR, miR-96 accelerated cell proliferation in BC cells. However, the accelerated cell growth mediated by miR-96 overexpression was not observed in cells co-overexpressing miR-96 and FOXO3a without 3′UTR indicating that miR-96 is regulating cell proliferation by targeting FOXO3a. Wu et al. (2012) has investigated the role of miR-370 and FOXO1 in human prostate cancer cells. In this study, they found that miR-370 expression level was upregulated in prostate cancer cell lines as compared to normal prostate epithelial cells and miR-370 directly targeted the FOXO1 3′UTR. miR-370 overexpression increased prostate cancer cell growth, and this effect of miR-370 on cell proliferation was decreased in the absence of FOXO1 3′UTR. These data suggest that miR-370 functions as an oncogenic factor by targeting FOXO1 in prostate cancer.

## REGULATION OF IGF-1 SIGNALING BY miRNAs IN MODEL ORGANISMS

In model organisms, some of miRNAs have been demonstrated to regulate IGF-1 signaling pathway. In *C. elegans*, miRNA lin-4 has been firstly reported to target lin-14 mRNA which is negative regulator of DAF-2. DAF-2 inactivation by lin-4 resulted in dampened IGF-1 signaling through the inhibition of DAF-16 (Boehm and Slack, 2005). miR-71 has been discovered to regulate lifespan of *C. elegans* through the deep sequencing (de Lencastre et al., 2010). In this study, PDK-1 expression was negatively regulated by miR-71 as a predicted target suggesting that miR-71 modulates lifespan via targeting the IGF-1 signaling pathway component (de Lencastre et al., 2010).

Hyun et al. (2009) has investigated the role of miRNAs in body size of *Drosophila* through the screening of cell proliferation-regulating miRNAs. They found miR-8 positively regulate body size targeting a fly gene named as u-shaped (*ush*) which is negative regulator of PI3K in fat body cells. In addition, they investigated human miR-200 which is homologous with miR-8 in *Drosophila* if it has also similar effect on PI3K-AKT activity. They found that miR-200 targeted FOG2 which binds to p85α and negatively regulate PI3K activity suggesting that miR-200 regulates cell proliferation by regulating PI3K-AKT signaling activity.

## THERAPEUTIC TARGETING OF miRNAs INVOLVED IN THE IGF-1 SIGNALING PATHWAY

miRNAs have the profound potential as therapeutic agents due to their unique properties. They regulate 100s of target genes, exerting proficient and synchronous post-transcriptional gene silencing effect for their targets (Chen et al., 2014). Their function can be easily manipulated by carefully designed oligonucleotides that lead to efficient and specific upregulation or downregulation of miRNAs (Li and Rana, 2014), referred to as miRNA mimics or antagomiRs, respectively. In addition, low molecular weight oligonucleotides are easier to deliver into the target cells compared with large viral vectors or plasmid normally used for gene therapy and are less likely to induce high immune response and toxicity as compared to plasmid DNA-based gene therapy and protein-based drug molecules (Chen et al., 2014). Therefore, miRNA-targeting therapies have been an active area of research, and many are already in preclinical and clinical development including HCV infection, different types of cancer, CVD, and insulin resistance (Li and Rana, 2014). miRNAs that regulate the IGF-1 signaling are prime targets for the development of novel therapies for several disease including cancer (**Table 1**). Indeed, let-7, a well-known tumor suppressor that regulates the multiple components of IGF-1 signaling pathway (**Table 1**), is currently targeted as a potential miRNA replacement treatment for cancer. Before the promising therapeutic impact of miRNA can be realized, there are several challenges to be addressed, which include but not limited to targeted delivery of oligonucleotides to specific organs, tissues and cell types, poor bioavailability, limited tissue permeability (Stylianopoulos and Jain, 2013), potential drug resistance, and instability (Raemdonck et al., 2008). Also, one of the biggest challenges is that miRNAs can induce the off-target silencing since miRNAs are designed to target multiple pathways *via* imperfect matching with 3′UTRs (Chen et al., 2014). The combination strategy can be applied to the miRNA therapy to minimize the unintended side effects and maximize the therapeutic effect (van Dongen et al., 2008).

## REGULATION OF miRNA EXPRESSION

Recently emerged question is how miRNAs are regulated under certain condition such as diseases. Transcriptional regulation of miRNAs has been reported in a few studies and it is one of the encouraged topics to be investigated for the role of miRNAs. miRNAs are classified into intergenic and intronic miRNAs by their genomic location. Intergenic miRNAs are transcribed by their own promoters while intronic miRNAs depend on the promoter of their host gene. Genome wide approaches have shown to match transcription factors to miRNA genes. For example, in mouse embryonic stem cells (ESCs), several pluripotency factors including Oct4 and Sox2 are associated with highly expressed miRNAs (Marson et al., 2008). Martinez et al. (2008) has reported transcription factors regulating miRNA expression in *C. elegans* using yeast one-hybrid (Y1H) assays. Recently, epigenetic modifications have been suggested as a mechanism of miRNA expression regulation. The atypical methylation of the CpG islands in the promoter regions both of intergenic and of intronic miRNAs has been involved in pathophysiology of various diseases (Chhabra, 2014). For example, miR-34 and miR-124 are most frequently hypermethylated in their promoter regions under pathological conditions while the promoter regions of let-7a-3 and miR-155 are hypomethylated in lung adenocarcinoma and B-cell lymphoma respectively (Costinean et al., 2006; Brueckner et al., 2007; Lujambio et al., 2007; Toyota et al., 2008; Wilting et al., 2010; Roy et al., 2012). These results may provide the clues to regulate miRNAs targeting IGF-1 signaling pathway.

## PERSPECTIVES

Regulation of IGF-1 signaling pathway has been an intense area of research in development of targeted therapeutics to improve human health. Ongoing clinical trials have focused on targeting protein components of IGF-1 signaling (Arcaro, 2013). For example, IGF-1R antibodies such as MK-0646 have been reported to be safe and reduce IGF-1R signaling in phase I and II clinical trials (Scartozzi et al., 2010; Reidy-Lagunes et al., 2012). In addition, several clinical trials in phase I and II of IGF-1R tyrosine kinase inhibitors such as OSI-906 are underway (Pitts et al., 2010). However, these agents have generated certain issues related to the different effects on signaling-reducing efficacy among patients (Arcaro, 2013). miRNAs and miRNA-targeting oligonucleotides offer promising therapeutic opportunities with several advantages over traditional small-molecule drugs. As the number of miRNAs targeting the IGF-1 signaling pathway increases, further progress will be made in miRNA-therapeutics to modulate many aspects of human disease through preclinical and clinical development.

## AUTHOR CONTRIBUTIONS

Hwa Jin Jung and Yousin Suh assembled relevant literature and wrote the manuscript.

## Conflict of Interest Statement

The authors declare that the research was conducted in the absence of any commercial or financial relationships that could be construed as a potential conflict of interest.
